# Identification of a Complex Karyotype Signature with Clinical Implications in AML and MDS-EB Using Gene Expression Profiling

**DOI:** 10.3390/cancers15215289

**Published:** 2023-11-04

**Authors:** Cheonghwa Lee, Ha Nui Kim, Jung Ah Kwon, Jinha Hwang, Ji-Ye Park, Ok Sarah Shin, Soo-Young Yoon, Jung Yoon

**Affiliations:** 1 Department of Laboratory Medicine, College of Medicine, Korea University, Seoul 08308, Republic of Korea; lunaaa03@gmail.com (C.L.); akikohatsu@naver.com (H.N.K.); jakwon83@korea.ac.kr (J.A.K.); jinha1226@gmail.com (J.H.); 2 BK21 Graduate Program, Department of Biomedical Sciences, College of Medicine, Korea University Guro Hospital, Seoul 08308, Republic of Koreaoshin@korea.ac.kr (O.S.S.)

**Keywords:** complex karyotype, AML, MDS, gene expression, SOD1

## Abstract

**Simple Summary:**

Complex karyotype (CK), defined as ≥3 unrelated chromosomal abnormalities, is associated with a poor prognosis in both acute myeloid leukemia (AML) and myelodysplastic syndrome (MDS). Despite their high genetic complexity, complex chromosomal abnormalities in AML and MDS may share dysregulated gene expression signatures, leading to poorer outcomes compared to those of standard chemotherapy. We aimed to investigate a subset of genes and their signatures to predict CK in AML and MDS with excess blast (MDS-EB) using gene expression data. The CK signature (CKS) was established and validated, and its prognostic impact on overall survival (OS) was evaluated in comparison with previously reported risk stratification models using gene expression. A 10-gene CKS demonstrated high predictive accuracy for CK and was associated with shorter OS with comparable performance to previously established risk stratification models.

**Abstract:**

Complex karyotype (CK) is associated with a poor prognosis in both acute myeloid leukemia (AML) and myelodysplastic syndrome with excess blasts (MDS-EB). Transcriptomic analyses have improved our understanding of the disease and risk stratification of myeloid neoplasms; however, CK-specific gene expression signatures have been rarely investigated. In this study, we developed and validated a CK-specific gene expression signature. Differential gene expression analysis between the CK and non-CK groups using data from 348 patients with AML and MDS-EB from four cohorts revealed enrichment of the downregulated genes localized on chromosome 5q or 7q, suggesting that haploinsufficiency due to the deletion of these chromosomes possibly underlies CK pathogenesis. We built a robust transcriptional model for CK prediction using LASSO regression for gene subset selection and validated it using the leave-one-out cross-validation method for fitting the logistic regression model. We established a 10-gene CK signature (CKS) predictive of CK with high predictive accuracy (accuracy 94.22%; AUC 0.977). CKS was significantly associated with shorter overall survival in three independent cohorts, and was comparable to that of previously established risk stratification models for AML. Furthermore, we explored of therapeutic targets among the genes comprising CKS and identified the dysregulated expression of superoxide dismutase 1 (*SOD1*) gene, which is potentially amenable to SOD1 inhibitors.

## 1. Introduction

Acute myeloid leukemia (AML) and myelodysplastic syndrome (MDS) are clonal disorders of myeloid hematopoiesis. The genetic signature of MDS, specifically MDS with excess blasts (MDS-EB), overlaps with that of secondary AML [1,2,3,4,5]. Similar cytogenetic abnormalities, such as del(5q), del(7q), and complex karyotypes (CK), have been reported in MDS and secondary AML, in contrast to de novo AML [6]. Gene mutations involved in the same pathways have been consistently identified in MDS and secondary AML [6], and these genetic signatures have helped refine MDS-type AML identification. Additionally, gene mutations highly specific for secondary AML have defined a distinct subset of de novo AML with clinical features and pathological properties of secondary AML [2]. Moreover, MDS-EB may be considered equivalent to secondary AML for therapeutic purposes [7].

CK, defined as ≥3 unrelated chromosomal abnormalities in both AML and MDS, comprises 10–12% of de novo AML and primary MDS cases [8,9]. CK is associated with a poor prognosis in both AML and MDS. In AML, CK is persistently associated with adverse outcomes and resistance to standard chemotherapy [10,11,12], suggesting that these entities are biologically distinct. Although a high frequency of *TP53* mutations is considered the hallmark of CK [13,14], other molecular features of CK, including the transcriptome, are less well characterized, possibly owing to its high genetic complexity.

Extensive transcriptomic analyses of myeloid neoplasms for the development of prognostic gene expression signatures have been beneficial in prognosis prediction and the detection of potential actionable targets [15,16,17,18,19,20,21]. However, transcriptomic analyses of prognostic gene expression signatures in myeloid neoplasms with specific cytogenetic abnormalities, particularly the CK subgroup, have rarely been conducted [17,18,19,20,21,22]. While the resolution of CK detection using gene expression signatures may be lower than conventional copy number variation detection methods such as SNP array, implementation of CK gene signature have the potential to add to the understanding of the impact of fusion transcript as well as the copy number variations associated with CK. 

Despite their high genetic complexity, complex chromosomal abnormalities in AML and MDS-EB may share dysregulated gene expression signatures, leading to poorer outcomes compared to those of standard chemotherapy. In this study, we aimed to investigate a subset of genes and their signatures to predict CK in AML and MDS-EB using gene expression data. Consequently, we integrated gene expression data from our cohort with the gene expression profiles of AML and MDS-EB with known karyotype results. The CK signature (CKS) was established and validated, and the prognostic impact of CKS on overall survival (OS) was evaluated in comparison with previously reported risk stratification models using gene expression. 

Additionally, to seek expanded utilization of CKS for future treatment selection, we conducted an exploration of therapeutic targets among the genes comprising CKS and identified the dysregulated expression of superoxide dismutase 1 (*SOD1*) gene, which is potentially amenable to SOD1 inhibitors.

## 2. Materials and Methods

### 2.1. Patient Samples

**Figure 1:**
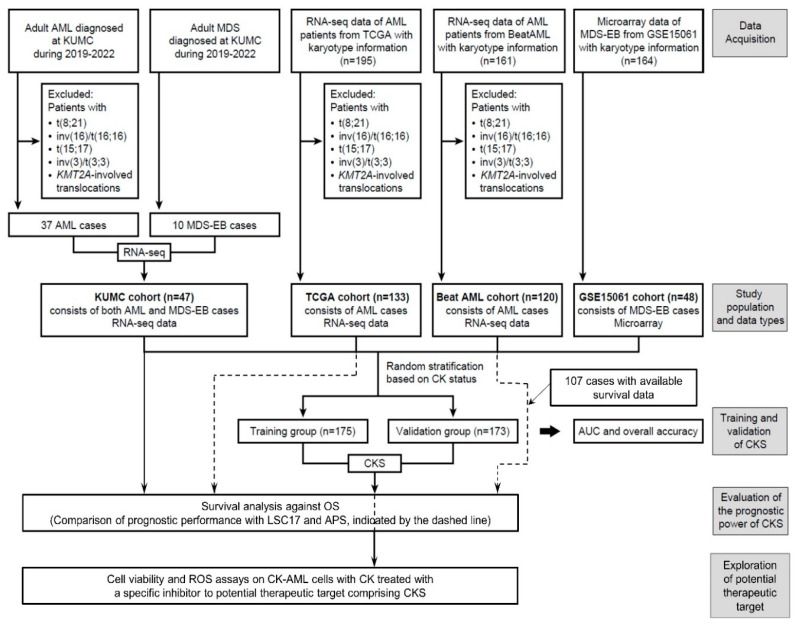
Overview of the study design. The flowchart explains the selection and filtering of the patient cohort in the model development, validation, and prediction of clinical outcome.

Our cohort (KUMC cohort) included 47 adults diagnosed with AML or MDS-EB. As the definition of CK in previous studies excludes WHO-designated balanced abnormalities, including t(8;21)(q22;q22.1), inv(16)(p13.1q22) or t(16;16)(p13.1;q22), t(15;17)(q22;q11–12), any translocations involving *KMT2A* [8,10,12,13], and inv(3)(q21.3q26.2) or t(3;3)(q21.3;q26.2) [8], AML patients with above abnormalities were excluded from our study (Figure 1). CK was defined as ≥3 unrelated chromosome abnormalities in both AML and MDS-EB. OS was defined as the time from diagnosis to death, with living patients censored on the date of their last follow-up. This study was approved by the Institutional Review Board of Korea University Guro Hospital (2021GR0572) and was conducted in accordance with the Declaration of Helsinki.

The therapeutic strategies applied to the patients in our cohort are as follows: The standard treatment regimen for AML patients most commonly (for 75.68% of AML patients) involved high-intensity cytarabine and idarubicin (7 + 3), or daunorubicin (including one case combined with midostaurin), followed by consolidation with high-dose cytarabine or low-intensity decitabine (for 24.32%, including one case combined with venetoclax). The salvage treatment regimen for the 37.84% of AML patients who did not respond to the initial induction consisted of Fludarabine, cytarabine, G-CSF, and idarubicin regimen for the majority of the cases (78.57%) and a venetoclax + decitabine regimen for the rest. In 8.11% of AML patients, salvage therapy was administered multiple times, involving alterations in the sequence of the previously mentioned regimen or the use of a regimen that included mitoxantrone, intermediate-dose cytarabine, and etoposide. Allogeneic peripheral blood stem cell transplantation (alloPBSCT) was performed in 21.62% of AML patients. The therapy for MDS-EB patients primarily consisted of 5-day decitabine or 7-day azacitidine, occasionally supplemented with low-dose cytarabine or alloPBSCT in 20% of patients, respectively.

To establish CKS, datasets from The Cancer Genome Atlas (TCGA) AML [23], Beat AML [24], and GSE15061 [25] were included. Clinical information, including chromosome analysis results and RNA sequencing (RNA-seq) data for the TCGA AML and Beat AML cohorts, was retrieved from cBioportal for Cancer Genomics, and gene expression data for GSE15061 were downloaded from the Gene Expression Omnibus database. For TCGA AML data, we validated ‘Cytogenetic abnormality type’ (for 195 cases where the data were available for CK classification), excluded WHO-designated balanced abnormalities (62 cases), and defined CK based on categorization (133 cases). For the Beat AML cohort, diagnostic bone marrow (BM) aspirate samples with RNA-seq and chromosome analyses data available for CK classification (161 cases) and not suspected of the exclusion criteria were analyzed (120 cases). For GSE15061, among the diagnostic BM samples of patients with MDS-EB (48 cases) in the MDS cohort (164 cases), CK was defined based on categorization. 

### 2.2. RNA Extraction, Library Preparation, and RNA-Seq

The diagnostic BM samples were subjected to RNA-seq analysis. RNA was extracted using the QIAamp RNA Blood Mini Kit (Qiagen, Venlo, The Netherlands) and reverse-transcribed to complementary DNA (cDNA) using the SuperScript VILO cDNA synthesis kit (Thermo Fisher Scientific, Waltham, MA, USA). RNA-seq libraries were prepared using the TruSeq Standard Total RNA LT Sample Prep Kit (Illumina, Inc., San Diego, CA, USA) and sequenced on the NovaSeq Sequencing system (Illumina, Inc.). Sequenced reads were aligned to the reference genome GrCh37 (hg19) using HISAT2 (version 2.1.0) [26], and aligned reads were estimated using StringTie (version.2.1.3b) [27]. 

### 2.3. Differential Gene Expression and Pathway Analysis 

Samples in the TCGA AML and Beat AML datasets were measured using the RNA-seq platform. RNA-seq data from the KUMC, TCGA AML, and Beat AML cohorts were subjected to differential gene expression analysis between patients with and without CK within each cohort using the DESeq2 package (version 1.32.0) [28]. The raw expression count data were used, and differentially expressed genes (DEGs) were identified using the threshold of adjusted *p* value < 0.05 and absolute log_2_ fold change > 0.5. The GSE15601 dataset included microarray data from the GPL570 platform (Affymetrix Human Genome U133 Plus 2.0 Array). DEGs between the CK and non-CK groups were identified using the limma R package [29], and the threshold of adjusted *p* value < 0.1 and absolute log_2_ fold change > 1.5 was applied. The DEGs that were common in at least two cohorts were selected. Overrepresentation analysis was performed using ConsensusPathDB (release 35) [30]. Pathway datasets included the Kyoto Encyclopedia of Genes and Genomes (KEGG), Reactome, and Wikipathways. 

### 2.4. Signature Development for CK Prediction

An overview of the study design and implementation is presented in Figure 1. We randomly classified our study population into training (*n* = 175; 35 patients with CK and 140 patients with non-CK) and validation (*n* = 173; 34 patients with CK and 139 patients with non-CK) cohorts stratified based on the CK status to generate a CK prediction model and then subsequently validate the model. For the KUMC, TCGA AML, and Beat AML cohorts, normalized values based on reads per kilobase per million mapped reads (RPKM) were used for downstream analysis. Quantile normalization was performed using the preprocessCore package (version 1.58.0). As for GSE15061, data processing was performed using the trimmed mean of differences between perfectly matched and mismatched intensities with the quantile normalization (DQN) algorithm [31]. For signature development, z-score transformation was performed on a per gene basis and calculated using z = (x − μ)/σ (where μ and σ are the gene mean and standard deviation, respectively) for cross-platform normalization, as described elsewhere [32]. To select a subset of genes from the training cohort for signature development, LASSO regression was applied using the glmnet package (version 4.1-4) [33] and validated using the leave-one-out cross-validation (LOOCV) method of the caret package (version 6.0-92) [34] to fit a logistic regression model. A subset of ten genes was selected, and the weighted combined gene expression (i.e., the CK score) was calculated to predict CK in the training cohort. Using the ‘predict’ function of the glmnet package, the patients were categorized as ‘CKS’ or ‘non-CKS’ at a threshold of 0.5. 

The values for the leukemic stem cell 17 signature score (LSC17) and AML prognostic score (APS) were calculated using model coefficients described by Ng et al. and Docking et al., respectively [17,18]. Briefly, LSC17 and APS were calculated as the sum of the log2-transformed RPKM-scaled counts weighted by the regression coefficients. Each cohort was dichotomized using their respective median scores to define high and low scores.

### 2.5. Cell Viability and ROS Assays on AML Cells with CK Treated with LCS-1, a Specific Inhibitor of Superoxide Dismutase 1 (SOD1)

Human AML HL-60 cells karyotyped as CK were purchased from the Korean Cell Line Bank (KCLB, Seoul, Republic of Korea) and maintained in RPMI 1640 (Corning Mediatech, Corning, NY, USA) supplemented with 10% fetal bovine serum (FBS; Corning Mediatech) and 25 mM HEPES at 37 °C in 5% CO_2_. HL-60 cells were seeded in 6-well plates and treated with LCS-1 (Merck, Darmstadt, Germany) at various time points. After incubation, the cells were collected and centrifuged at 300× *g* for 10 min. The cell pellet was washed with cold Dulbecco’s phosphate-buffered saline (DPBS) and homogenized using an Ultra-Turrax T8 homogeniser (IKA-Werke GmbH&Co. KG, Staufen, Germany). Thereafter, the samples were centrifuged at 300× *g* for 10 min, and the supernatant was used to measure SOD enzymatic activity using a SOD Colorimetric Activity Kit (Invitrogen). The assay was conducted according to the manufacturer’s protocol, and the unit of SOD activity was calculated relative to the standard reagent provided in the kit.

For cell viability assay, HL-60 cells were seeded in 96-well plates and treated with various concentrations (0, 0.1, 0.5, 1, and 2.5 μM) of LCS-1 diluted in serum-free RPMI 1640 for 24 h. After incubation, a thiazolyl blue tetrazolium bromide (MTT) solution (Sigma-Aldrich, Burlington, MA, USA) was added to each well of the plate and incubated at 37 °C for 4 h. Subsequently, dimethyl sulfoxide (Sigma-Aldrich) was added to each well and incubated for 0.5 h. The absorbance at 450 nm was measured using Varioskan LUX multimode microplate reader (Thermo Fisher Scientific). The control was set to 100%, and the absorbance of the other samples were calculated relative to the control. In addition, HL-60 cells were stained with 5 μg/mL Propidium iodide (PI) (Sigma-Aldrich) for 20 min and washed with DPBS. The stained cells were analyzed using the EVOS™ FL Auto 2 imaging system (Thermo Fisher Scientific). PI fluorescence was quantified using ImageJ software (version 1.52a), and each treated sample was calculated relative to the control samples.

Intracellular reactive oxygen species (ROS) was measured using 2′,7′-dichlorofluorescin diacetate (DCFDA, Sigma-Aldrich), which is a cell-permeable, non-fluorescent probe that exhibits green fluorescence upon oxidation. Mitochondrial ROS was measured using MitoSOX™ (Invitrogen, Carlsbad, CA, USA), a fluorescent dye specific for detecting mitochondrial superoxide production in cells. Briefly, the cells were treated with 1 μM DCFDA or 5 μM MitoSOX™ in serum-free medium and incubated for 20 min at 37 °C. The cells were then washed with DPBS. The cells stained with fluorescent dyes were detected using an EVOS™ FL Auto 2 Imaging system (Thermo Fisher Scientific). The fluorescence values were quantified using ImageJ software, and each condition sample was calculated relative to the control samples.

### 2.6. Statistical Analysis

All statistical analyses and visualizations were performed using the R software (version 3.4.3). For model validation using LOOCV, the receiver operating characteristic (ROC) curves and the area under the curve (AUC) were analyzed. For analysis of patient characteristics, comparisons between the two groups were performed using the Kruskal–Wallis rank sum test for continuous variables and the Fisher’s exact test for categorical variables. Survival was estimated using the Kaplan–Meier method, log-rank test, and Cox proportional hazard regression with the Survminer (version 3.4.3) package.

## 3. Results

In our cohort (KUMC cohort), a total of 47 patients with AML or MDS-EB (AML/MDS-EB) were included. The main clinical and hematological characteristics of the patients are presented in Table 1. CK, defined as ≥3 unrelated chromosomal abnormalities, was detected in 16 patients with AML/MDS (10 AML and 6 MDS-EB), with a mean number of chromosomal abnormalities of 15. The degree of karyotype complexity was not significantly different between AML with CK and MDS-EB with CK in our study (*p* = 0.3855). 

The diagnosis distribution within the KUMC cohort following the introduction of the International Consensus Classification (ICC) 2022 is presented in Supplementary Table S1.

### 3.1. Differential Gene Expression Profile between CK and Non-CK Group

The gene expression data of KUMC, TCGA AML, Beat AML, and GSE15061 were subjected to differential gene expression analysis between patients with and without CK within each cohort, including 69 patients with CK and 279 patients with non-CK. DEGs common in at least two of the four cohorts were selected. A total of 404 DEGs were identified, including 222 upregulated and 182 downregulated genes (Supplementary Table S2).

**Figure 2:**
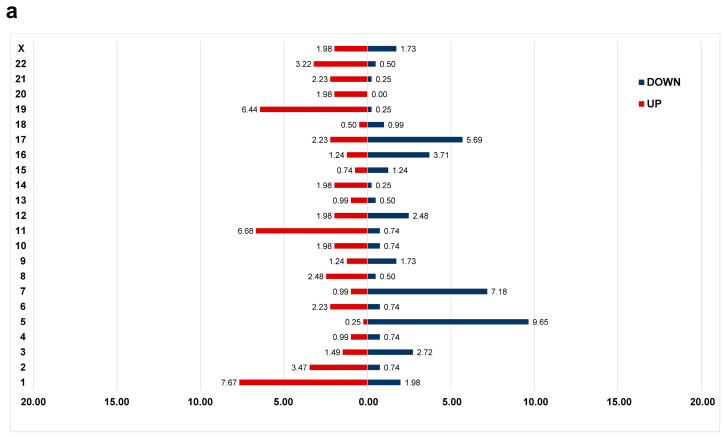

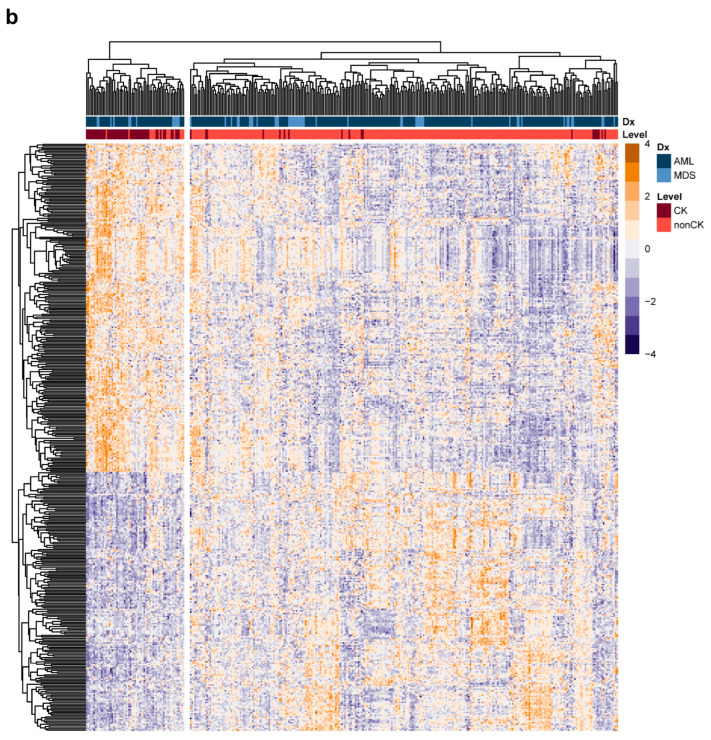

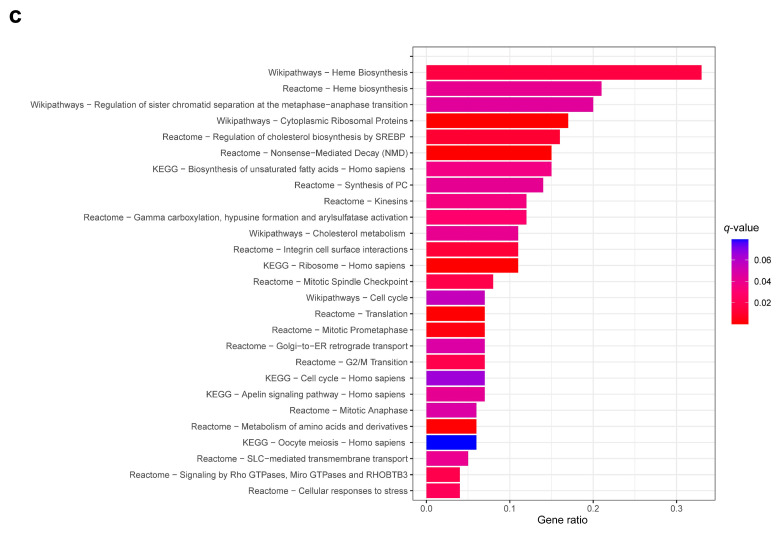
Differential gene expression profiles between the complex karyotype (CK) and non-CK group. (**a**) The distribution of differentially expressed genes (DEGs) in the CK group compared to that of the non-CK group based on chromosome location. The y-axis represents chromosome numbers, and the x-axis indicates the proportion of DEGs located on each chromosome out of the total DEGs. The red bars represent the fraction of upregulated genes among all DEGs (a total of 0.55), whereas the blue bars represent the fraction of downregulated genes (a total of 0.45). (**b**) A heatmap of 404 DEGs between the CK and non-CK groups. (**c**) The over-representation analysis based on Reactome, Wikipathways, and KEGG databases. The fraction of genes belonging to each term out of total listed genes is shown on the *x*-axis, and the q-values of ≤ 0.05 are considered significant and plotted accordingly.

Notably, the downregulated genes were mostly localized on chromosome 5 or chromosome 7 and the upregulated genes were localized on chromosome 1 or chromosome 11 (Figure 2a). When examining the localization of DEGs by chromosome arm, the majority of downregulated genes were found on the long arm of either the chromosome 5 (*n* = 38, 9.41% of DEGs), chromosome 7 (*n* = 20, 4.95% of DEGs), or chromosome 17 (*n* = 17, 4.21% of DEGs). More than half of the DEGs located on the 17q were distributed in the 17q21 band (*n* = 9), while on chromosomes 5 and 7, DEGs were distributed throughout the long arm. For upregulated genes, 20 genes each (4.9% of DEGs) were located on the long arm of chromosome 11 and short arm of chromosome 1, respectively, and were most frequently observed in the 11q13 (*n* = 7) and 1p34 (*n* = 8) bands. 

Hierarchical clustering of the 404 DEGs revealed the possibility of differentiating between the CK and non-CK groups based on gene expression patterns (Figure 2b). Overrepresentation analysis revealed the dysregulation of genes significantly involved in cell cycle (e.g., *BUB1, ORC1, YWHAH, ESPL1, WEE1, CDC20, TFDP1, CHEK1*), heme biosynthesis (*UROD, HMBS, UROS*), and cellular responses to stress (e.g., *RPS14, RPL26, FBXL17, SOD1*) (Figure 2c, Supplementary Table S3). Dysregulation of several genes associated with chromosome instability, such as *BUB1* and *CDC20* were noted in CK group [35,36]. Upregulated DEGs were significantly enriched in cell cycle, heme biosynthesis, whereas downregulated DEGs were significantly enriched in ribosome and cholesterol metabolism. 

### 3.2. CKS Training and Validation

**Figure 3:**
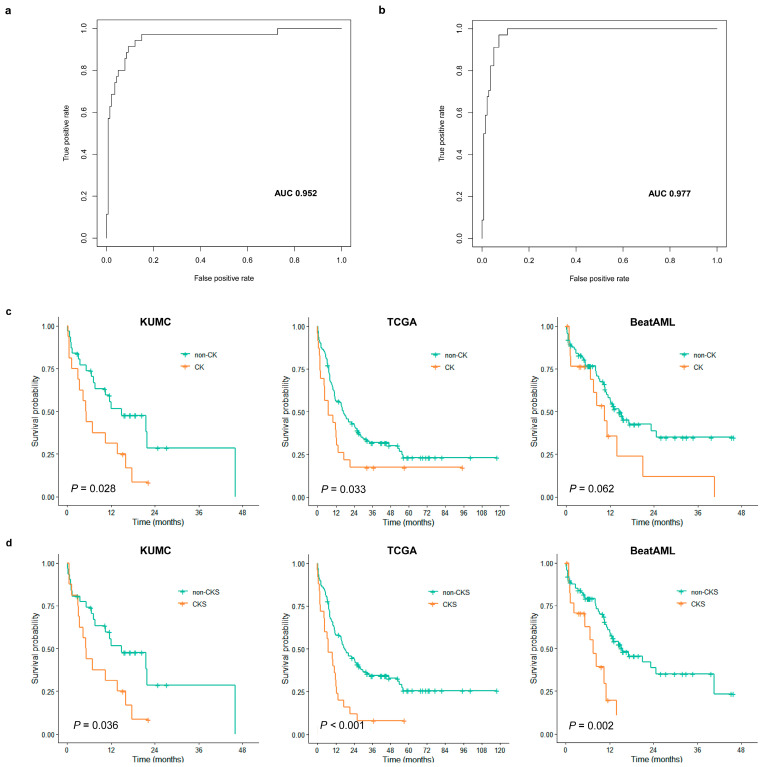
Evaluation results of complex karyotype signature (CKS) classification performance and prognostic impact. LOOCV-estimated AUC of CKS score for predicting CK in the training cohort (**a**) and validation cohort (**b**). (**c**) Kaplan–Meier curves against overall survival (OS) according to CK (orange line) and non-CK (green line) in KUMC (left), TCGA (middle), and BeatAML (right) cohorts. (**d**) Kaplan–Meier curves against OS according to predicted as CK by CKS (orange line) and non-CKS (green line) in KUMC (left), TCGA (middle), and BeatAML (right) cohorts.

Based on the differential gene expression profiles of the CK group, we investigated whether the expression data from 404 DEGs could be used to establish a more robust CKS for CK classification. To establish the CKS gene set for predicting CK, the final set of ten DEGs were extracted using LASSO regression to fit a multivariate logistic regression model. The model development process using the training cohort defined the CKS calculated for each patient using gene expression weighted by regression coefficients as follows: CKS score = −6.157 + (*PRX* × 1.7021) + (*MMD* × 1.5629) + (*KIAA1549* × −1.2916) + (*GIPC1* × 0.9794) + (*RAB33A* × 2.1416) + (*SOD1* × 2.1254) + (*MED7* × −1.8714) + (*COPG2* × −2.0169) + (*RPS14* × −1.204) + (*PSMB10* × −1.6775). The CKS score in the training cohort showed an LOOCV-estimated AUC of 0.952 (Figure 3a), with an overall accuracy of 95.43% (*n* = 167/175) for CK prediction. As the CKS scores differed significantly between the CK and non-CK groups (*p* < 0.0001), we further evaluated the CKS model using the validation cohort. In the validation cohort, the AUC was 0.977 (Figure 3b), with an overall accuracy of 94.22% (*n* = 161/173), and the CKS score was sufficient to differentiate between the CK and non-CK groups (*p* < 0.0001).

### 3.3. Assessment of CKS Prediction for CK

An analysis was conducted on cases where there were discrepancies in the prediction of CKS of CK. The proportion of inaccurate predictions showed no difference between MDS-EB and AML group (6.8% vs. 5.5%), demonstrating that the CKS was capable of predicting CK in patients with AML and MDS-EB.

There were ten non-CK cases with a CK gene expression signature, resulting in a specificity of 96.42%. No differences were observed in terms of clinical outcome from a comparison between these commonly referred to as false positive cases and true negative cases. The most common unbalanced chromosomal abnormalities in CK involve the loss of 5q, 7q and 17p [13,14]. Notably, among the genes comprising CKS, the four out of the five downregulated genes are located on 5q and 7q. Therefore, we examined the association between false positive cases and these deletions in cases with karyotype information (Supplementary Table S4). 5q deletion and 7q deletion were observed in one sample each, however, such deletion was not accompanied in the majority of cases, drawing a generalized conclusion remains premature. Among four samples for which microarray data were available, one sample exhibited complexity corresponds to CK (copy-number alterations of more than 5 Mbp in more than three different chromosome arms).

Eight CK cases were identified as non-CK by CKS, resulting in a sensitivity of 88.40%. However, these false negative cases in TCGA and BeatAML cohorts had significantly longer overall survival compared to accurately predicted positive cases (TCGA: median OS of 64.0 vs. 7.0, *p* = 0.032; BeatAML: 20.9 vs. 5.0, *p* = 0.010). The discrepancy that compromised sensitivity could reflect cases with more favorable clinical outcomes. In the KUMC cohort, there was a single instance of a false negative which precluded meaningful statistical analysis. Among the six samples with available karyotype information, a subclone of the CK clone was observed in only two to three metaphases out of the entire metaphase population in two thirds of these samples (Supplementary Table S4). This underscores a potential limitation of RNA-seq, which, being a bulk analysis, may not fully capture subclones that constitute a small fraction of the population. The two samples for which microarray data were available showed low complexity corresponding to non-CK.

### 3.4. Evaluation of CKS as a Prognostic Score

We evaluated the prognostic impact of the CKS determined using the CKS score in predicting clinical outcomes. Three cohorts with survival data, namely, the KUMC, TCGA AML, and Beat AML cohorts, were analyzed. In the KUMC and TCGA cohorts, all patients included in the CKS model development were also included in the survival analysis, whereas 107 patients (CK group *n* = 89; non-CK group *n* = 18) with available survival data were included in the Beat AML cohort. 

Patients with CK in the KUMC cohort were characterized by lower white blood cell (WBC) counts (*p* = 0.021; median value of 2.6 vs. 5.4 × 10^9^/L) (Table 2). CK was significantly associated with shorter OS; the estimated 1-year OS for patients with CK was 31.2% and that for patients without CK was 55.6% (*p* = 0.028) (Figure 3c). Survival analysis of CKS also revealed a significant association with shorter OS (*p* = 0.0360; 1-year OS, 31.2% vs. 55.7%) (Figure 3d). We performed univariate Cox analysis with stratified patient age, peripheral blood WBC count, diagnostic BM blast count, presence of *FLT3*-ITD, *RUNX1*, presence of CK, and three dichotomized prognostic models, including CKS, LSC17, and APS. In the KUMC cohort, CK and CKS were identified as the sole predictors of OS with a strong association (CK: HR = 2.1812, *p* = 0.0318; CKS: HR = 2.1098, *p* = 0.0402) (Table 3).

We then investigated the clinical impact of CK and CKS on the TCGA AML and Beat AML cohorts. In both cohorts, lower WBC counts and lower BM blast percentages were observed in the CK group, as in the KUMC cohort (Supplementary Tables S5 and S6). CK was significantly associated with shorter OS in the TCGA AML cohort (*p* = 0.033; 1-year OS, 56.1% vs. 39.1%), and a trend toward shorter OS in the Beat AML cohort (*p*= 0.062; 1-year OS, 56.54% vs. 35.7%) (Figure 3c), whereas CKS was significantly associated with shorter OS in both cohorts (TCGA AML: *p* = 0.00043; 1-year OS, 58.1% vs. 32.0%; Beat AML: *p* = 0.0015; 1-year OS, 59.1% vs. 19.6%) (Figure 3d). In both cohorts, based on the univariate Cox analysis, an association or resembling trend between CK and OS was noted (TCGA AML: HR = 1.7175, *p* = 0.0353; Beat AML: HR = 1.8371, *p* = 0.0657), and a stronger association was observed with CKS (TCGA AML: HR = 2.2926, *p* = 0.0006; Beat AML: HR = 2.8349, *p* = 0.0024) (Table 3). Among clinical variables, only stratified patient age was found to affect OS. In the multivariate analysis incorporating this variable, CKS retained its strong independent prognostic power in both cohorts (TCGA AML: adjusted HR = 1.8671, *p* =0.0121; Beat AML: adjusted HR = 2.6512, *p* = 0.0050) (Table 3).

### 3.5. Comparison of Prognostic Performance with Other Models

As the prognostic impact of CKS was evaluated, we further performed a comparison between CKS and previously developed models, namely, LSC17 score and APS, in the prediction of outcomes in patients with AML. The LSC17 score is a model based on the expression of 17 leukemic stem cell-enriched genes and accurately predicts poor prognosis in patients with AML [18,19,37,38]. APS is a 16-gene expression signature model developed to improve risk stratification of patients with AML [17]. These models were established to predict the prognosis of all patients with AML, including those with WHO-designated balanced abnormalities. Prognostic prediction in the context of specific cytogenetic abnormalities, such as CK, has not been previously evaluated. We calculated the LSC17 score and APS for each AML sample in the TCGA AML and Beat AML cohorts and observed that samples with above-median scores (within each cohort) had significantly worse survival outcomes in the TCGA AML (LSC17: HR = 1.7483, *p* = 0.0068; APS: HR = 1.4957, *p* = 0.0486) and Beat AML cohorts (LSC17: HR = 2.1387, *p* = 0.0097; APS: HR = 2.2187, *p* = 0.0065) (Table 3). However, in each cohort, the hazard ratio for CKS was higher than that for both LSC17 and APS in univariate Cox analysis. When included as covariates in the multivariate Cox analysis, the CKS retained a higher hazard ratio than the above two prognostic models in both cohorts (Table 3). 

### 3.6. Effects of SOD1 Inhibition on AML Cell Proliferation and ROS Production

To investigate the feasibility of utilizing CKS for therapeutic selection, we have explored the potential of genes constituting CKS as prospective therapeutic targets. Through a literature review, we have observed that *SOD1*, one of the components of CKS, is overexpressed in the CK group and is associated with the development of various types of cancers [39,40]. As an antioxidant enzyme, SOD1 is also a part of the cellular response to stress pathway, which our overrepresentation analysis revealed to be dysregulated in the CK group.

**Figure 4:**
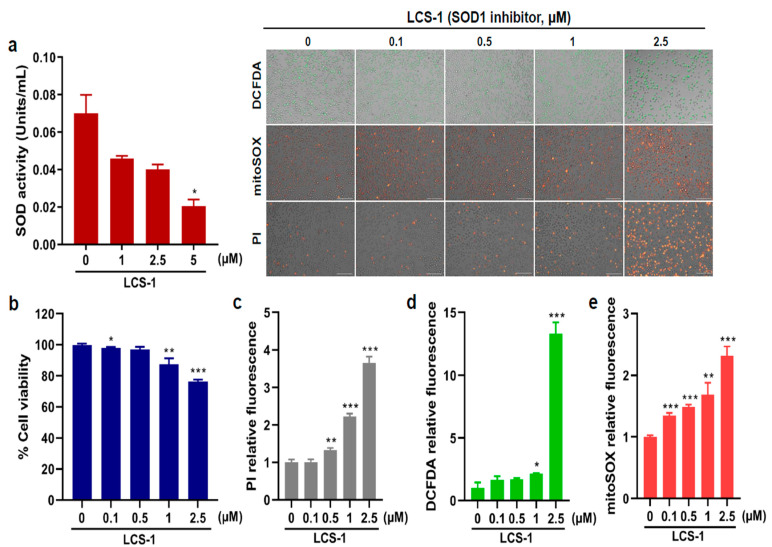
SOD1 inhibitor treatment leads to reduction in AML cell proliferation. (**a**) Superoxide dismutase (SOD) enzymatic activity (units/mL) measured after treatment with various concentrations (0, 1, 2.5, and 5 μM) of LCS-1 in HL-60. (**b**) Cell viability (%) measured after treatment with different concentrations (0, 0.1, 0.5, 1, and 2.5 μM) of LCS-1. (**c**) The production of dead cells detected by fluorescence intensity measured after treatment with LCS-1 and staining with PI. (**d**) The production of intracellular reactive oxygen species (ROS) detected by fluorescence intensity measured after treatment with LCS-1 and staining with DCFDA. (**e**) The production of mitochondrial ROS detected by fluorescence intensity measured after treatment with LCS-1 and staining with mitoSOX. The concentrations of LCS-1 used for (**c**–**e**) are the same as the concentrations used in b. Bar graphs show mean ± SD of fluorescence intensity quantified by ImageJ. Scale bar represents 100 μm. Statistical analysis: * *p* < 0.05; ** *p* < 0.01; *** *p* < 0.001 vs. untreated control cells.

To determine if specific SOD1 inhibition would affect the cellular proliferation of the AML cells HL-60, we treated these cells with the specific SOD1 inhibitor LCS-1. As shown in Figure 4a, LCS-1 suppressed SOD activity in a dose-dependent manner. To further examine whether LCS-1 affects HL-60 viability, we performed cell viability assays, which showed that SOD1 inhibition decreased cell viability in HL-60 (Figure 4b). Consistent with the inhibitory effect of LCS-1 on cell viability, LCS-1 treatment led to a higher number of dead cells in the treatment group than in the control group (Figure 4c). Given that SOD1 plays an essential role as an antioxidant enzyme, we investigated whether LCS-1 affects ROS production. As shown in Figure 4d,e, SOD1 suppression caused a marked increase in total as well as mitochondrial ROS levels in HL-60. These findings show that SOD1 inhibition induces AML cell apoptosis and ROS production, suggesting the potential of SOD1 as a therapeutic target.

## 4. Discussion

CK is associated with a poor prognosis and resistance to standard chemotherapy in both AML and MDS-EB [10,11,12]. In this study, we developed a 10-gene expression model to predict CK expression in AML and MDS-EB. The CKS was capable of accurately predicting CK in both the training and validation cohorts. The CKS demonstrated a prognostic impact, as the CKS score is a hazard indicator of magnitude similar to the previously reported risk stratification models, LSC17 and APS. 

In accordance with prior studies of the most frequently reported chromosome arms of genomic imbalances (losses and gains) in CK-AML patients, the losses were reported with following regions and frequencies: 5q, 17p, 7q, 18q, 16q, and 17q and conversely, gains, while less prevalent than deletions, were reported with following frequencies: 8q, 11q, 21q, 22q, 1p, and 9p [39]. In this study, downregulated genes were notably found on 5q, 7q, and 17q, while upregulated genes were identified on 11q and 1p, showing high degree of alignment with previous findings. 

The disease mechanism of CK is currently not fully elucidated; however, in MDS or AML with 5q and/or 7q deletions, haploinsufficiency of genes in the deleted region is considered one of the prevailing mechanisms resulting in disease development and malignant progression. DEGs localized at 5q or 7q were previously suggested to be putative haploinsufficient genes based on the location of genes in the commonly deleted region (5q: *FBXL17, PCBD2, TIGD6;* 7q: *TPK1, ZCWPW1*) and/or gene expression (5q: *HINT1, FAM13B, PCBD2, RPS14*; 7q: *GSTK1, TBXAS1, ZNF227*) [40,41,42,43,44,45]. The gene expression profile of CK in this study suggests that multiple haploinsufficiencies due to the deletion of 5q or 7q likely underlie the pathogenesis of CK, with some functionally related genes identified. For instance, *RPS14* has been implicated in the pathogenic mechanisms of cytopenias, including MDS with 5q deletion and congenital macrocytic anemias [46]. In addition, upregulation of DEGs localized at 11q and 1p may result from the duplication or amplification of 11q and 1p, previously observed in association with MDS or AML in patients with CK [39,47,48]. However, the pathogenic mechanisms as well as triplosensitivity of genes located on 11q and 1p are not as clear as in the cases of losses.

The treatment strategy for high-risk AML patients with CK primarily involves intensive chemotherapy, followed by allogeneic hematopoietic stem cell transplantation. Nevertheless, over 50% of CK-AML patients experience disease relapse, necessitating the exploration of novel treatment [49]. One of the previous studies highlighted on dysregulated pathways, demonstrating the disruption of cell cycle in CK, albeit limited to subgroups with *TP53* alterations and inhibitors targeting this pathway have shown promise in vitro and in vivo [50]. Our overrepresentation analysis also revealed the significance of the cell cycle, particularly the chromatin separation pathway in CK patients, aligning with these findings. In this regard, we further aimed to identify other novel pathway-related therapeutic targets for the CK group. Moreover, since CKS has proven valuable in risk-stratifying AML and MDS-EB patients, identifying genes within the CKS model that can also serve as therapeutic targets, could extend its application to predicting susceptible patients for targeted therapy in the future. During our investigation of the genes comprising the CKS model, we noticed that *SOD1*, which is overexpressed in the CK group, is also part of the dysregulated pathway identified through overrepresentation analysis—specifically, the cellular stress response pathway. 

*SOD1* codes for Cu/Zn superoxide dismutase associated with intracellular ROS regulation and is regulated by NF-E2 related factor (Nrf2). The interference with the Nrf2 pathway has been identified as one of the mechanisms of action of the Bcl-2 inhibitor venetoclax, which is currently used for the treatment of AML patients who are not eligible for intensive induction chemotherapy in combination with hypomethylating agents. Overexpression of Nrf2 has been linked to venetoclax resistance [51,52,53]. Targeting SOD1 may have implications for inhibiting the growth and proliferation of leukemia cell lines with CKs as well as resistance to drugs such as venetoclax. 

SOD1 has been reported in various human cancer types [54,55] and targeting SOD1 showed potential of inhibition of cancer growth in in vitro [56,57] and in vivo studies [58]. Regarding AML, *SOD1* overexpression has been associated with an adverse prognosis [59], and indirect inhibition of SOD1 resulted in selective apoptosis of leukemia cells [60]. We have demonstrated that SOD1 inhibition in CK-AML cell lines induces apoptosis and triggers ROS production. When combining our findings with existing research on leukemia cell lines [60], in becomes evident that SOD1 is an attractive candidate for further investigation as a therapeutic target for CK patients. Exploring the potential benefits of combining conventional treatments or agents targeting recently suggested therapeutic targets, such as *PLK1*, with SOD1 inhibitors could serve as a promising avenue for future research [50].

Owing to the relatively small proportion of patients with MDS-EB included in our study (16.67% of all patients included), it is possible that CKS may not fully encompass the molecular genetic characteristics of MDS-EB, even though hierarchical clustering of the 404 DEGs could not differentiate between MDS-EB and AML based on the gene expression patterns and the proportion of inaccurate predictions showed no difference between MDS-EB and AML group (6.8% vs. 5.5%, respectively). Further application of this formula to a larger MDS-EB group in the future may help validate its efficacy. Another limitation is that the cohort of patients used in this study was composed of diverse patient groups who received various treatments. Therefore, further studies are required to ensure the use of the CKS score for predicting the clinical outcome in the patient group using specific treatment regimens. Lastly, our cell culture was conducted targeting a single gene in a single cell line. While we have presented the potential for SOD1 inhibition following existing research [60], further validation is necessary with different cell lines. Additionally, a comprehensive consideration for the other genes comprising CKS as the candidate for therapeutic target is required.

## 5. Conclusions

Conclusively, we performed gene expression analysis of patients with AML and MDS-EB with CK and developed a model to predict CK. The significance of this study lies in its ability to diagnose CK using gene expression profiling. The results of this study imply that previously established haploinsufficiency due to the deletion of 5q or 7q possibly underlies the pathogenesis of CK. Moreover, we observed that the CKS could be used as a model to unravel potential therapeutic targets such as SOD1.

## Figures and Tables

**Table 1:** Patient characteristics of the KUMC cohort.

	KUMC (*n* = 47)
Sex (%)	
Male	29 (61.7)
Female	18 (38.3)
Age (years)	
Median (range)	67.0 (31.0–86.0)
Hb (g/dL)	
Median (range)	7.9 (5.9–12.1)
WBC count (×10^9^/L)	
Median (range)	3.8 (0.6–247.2)
Platelet count (×10^9^/L)	
Median (range)	51.0 (3.0–240.0)
BM blasts (%)	
Median (range)	40.0 (0.8–97.4)
Diagnosis	
AML with recurrent genetic abnormalities	17 (36.2)
AML with mutated *NPM1*	8 (17.0)
AML with biallelic mutation of *CEBPA*	3 (6.4)
AML with mutated *RUNX1*	6 (12.8)
AML with MRC	11 (23.4)
AML, NOS	9 (19.1)
MDS-EB	10 (21.3)
Risk stratification by 2022 ELN	
Favorable	7 (18.9)
Intermediate	9 (24.3)
Adverse	21 (56.8)
Risk stratificaton by IPSS-M	8 (17.0)
High	2 (20.0)
Very high	80 (80.0)
Number of chromosome abnormalities	
Mean ± SD	5.2 ± 8.2

**Table 2:** Patient characteristic of the KUMC cohort evaluated for clinical significance of complex karyotype.

	Total (*n* = 47)	CK (*n* = 16)	Non-CK (*n* = 31)	*p*
Sex (%)				0.691
Male	29 (61.7)	11 (68.8)	18 (58.1)	
Female	18 (38.3)	5 (31.2)	13 (41.9)	
Age (years)				0.661
Median (range)	67.0 (31.0–86.0)	66.0 (31.0–84.0)	67.0 (46.0–86.0)	
Hb (g/dL)				0.875
Median (range)	7.9 (5.9–12.1)	8.1 (6.5–9.7)	7.8 (5.9–12.1)	
WBC count (× 10^9^/L)				0.021
Median (range)	3.8 (0.6–247.2)	2.6 (1.4–30.4)	5.4 (0.6–247.2)	
Platelet count (× 10^9^/L)				0.148
Median (range)	51.0 (3.0–240.0)	34.0 (9.0–240.0)	56.0 (3.0–177.0)	
BM blasts (%)				0.116
Median (range)	40.0 (0.8–97.4)	26.5 (0.8–96)	48.1 (11.0–97.4)	
Risk stratification by genetics				
*FLT3-ITD*				0.104
without	40 (85.1)	16 (100.0)	24 (77.4)	
with	7 (14.9)	0 (0.0)	7 (22.6)	
*RUNX1*				0.155
without	39 (83.0)	16 (100.0)	23 (74.2)	
with	8 (17.0)	0 (0.0)	8 (25.8)	
Overall survival (months)				
Median (range)	10.3 (0.1–46.1)	5.1 (0.2–22.3)	11.6 (0.1–46.1)	
	AML Total (*n* = 37)	CK (*n* = 27)	Non-CK (*n* = 10)	
Risk stratification by 2022 ELN				0.005
Favorable	7 (18.9)	0 (0.0)	7 (25.9)	
Intermediate	9 (24.3)	0 (0.0)	9 (33.3)	
Adverse	21 (56.8)	10 (100.0)	11 (40.7)	

**Table 3:** Univariate and multivariate Cox regression analyses of the potential factors influencing overall survival. The figures displayed in the multivariate analysis represent the HR and *p*-values for CK, CKS, LSC17, and APS when co-analyzed with a factor that has been demonstrated to have a significant association with overall survival in preceding univariate analysis, namely stratified age.

Cohort	KUMC			TCGA			BeatAML		
Univariate Analysis									
	HR (95% CI)	*p*		HR (95% CI)	*p*		HR (95% CI)	*p*	
Age (>65 years)	1.1584 (0.5548–2.4185)	0.6969		2.8149 (1.8637–4.2516)	<0.0001		2.7475 (1.5945–4.7343)	0.0003	
PB WBC count	1.0000 (1.0000–1.0000)	0.9775		1.0026 (0.9984–1.0068)	0.2347		1.0002 (0.9960–1.0045)	0.9114	
BM blast count	0.9970 (0.9849–1.0093)	0.6364		0.9950 (0.9849–1.0053)	0.3428		0.9948 (0.9851–1.0046)	0.2978	
*FLT3-ITD* mutation	0.9978 (0.3839–2.5937)	0.9965		1.0012 (0.5922- 1.6927)	0.9963		1.8201 (0.9591–3.4542)	0.0683	
*RUNX1* mutation	1.6715 (0.5755–4.8550)	0.3475		1.4898 (0.8118–2.7339)	0.2004		1.2844 (0.6556–2.5161)	0.4680	
CK	2.1812 (1.0741–4.4293)	0.0318		1.7175 (1.0407–2.8343)	0.0353		1.8371 (0.9644–3.4994)	0.0657	
CKS	2.1098 (1.0376–4.2902)	0.0402		2.2926 (1.4286–3.6793)	0.0006		2.8349 (1.4498–5.5433)	0.0024	
LSC17	-	-		1.7483 (1.1692–2.6144)	0.0068		2.1387 (1.2059–3.7931)	0.0097	
APS	-	-		1.4957 (1.0045–2.2271)	0.0486		2.2187 (1.2529–3.9290)	0.0065	
Multivariate analysis									
	HR (95% CI)	*p*	Overall *p*	HR (95% CI)	*p*	Overall *p*	HR (95% CI)	*p*	Overall *p*
CK + Age	-	-	-	1.5819 (0.9562–2.6170)	0.0756	<0.0001	2.0341 (1.0605–3.9017)	0.0335	0.0003
CKS + Age	-	-	-	1.8671 (1.1495–3.0326)	0.0121	<0.0001	2.6512 (1.3458–5.2227)	0.0050	<0.0001
LSC17 + Age	-	-	-	1.5503 (1.0332–2.3262)	0.0351	<0.0001	2.0246 (1.1371–3.6047)	0.0171	0.0001
APS + Age	-	-	-	1.5428 (1.0360–2.2975)	0.0337	<0.0001	1.8607 (1.0336–3.3496)	0.0394	0.0003

## Data Availability

The data presented in this study are available on request from the corresponding author. The data are not publicly available due to privacy.

## Supplementary Materials

The following supporting information can be downloaded at: https://www.mdpi.com/article/10.3390/cancers15215289/s1, Table S1: Diagnosis of the KUMC patients according to 2022 ICC; Table S2: List of 404 differentially expressed genes; Table S3: Pathways identified in overrepresentation analysis; Table S4: List of discrepant cases in the prediction of CKS of karyotype with karyotype and microarray information; Table S5: Patient characteristic of the TCGA cohort evaluated for clinical significance of complex karyotype; Table S6: Patient characteristic of the BeatAML cohort evaluated for clinical significance of complex karyotype.
