# Supplementary material for: Identification of a Complex Karyotype Signature with Clinical Implications in AML and MDS-EB Using Gene Expression Profiling
Source: Cancers (Basel). 2023 Nov 4;15(21):5289. doi: 10.3390/cancers15215289 (PMC10648390; doi:10.3390/cancers15215289)
Supplement: Supplementary file 1 [file cancers-15-05289-s001.zip › Table S1.pdf]

Table S1. Diagnosis of the KUMC patients according to 2022 ICC.

	KUMC (n=47)
AML with recurrent genetic abnormalities	11 (23.4)
AML with mutated *NPM1*	8 (17.0)
AML with in-frame bZIP mutated *CEBPA*	3 (6.4)
AML with mutated *TP53*	8 (17.0)
AML with MR gene mutation	11 (23.4)
AML with MR cytogenetic abnormalities	1 (2.1)
AML, NOS	5 (10.6)
MDS/AML with mutated *TP53*	1 (2.1)
MDS/AML with MR gene mutation	4 (8.5)
MDS/AML with mutated *TP53*	3 (6.4)
MDS with mutated *TP53*	3 (6.4)

ICC International Consensus Classification, *AML* Acute myeloid leukemia, *MR* myelodysplasia-related, *NOS* not otherwise specified *MDS* Myelodysplastic syndrome.
