# Supplementary material for: Identification of a Complex Karyotype Signature with Clinical Implications in AML and MDS-EB Using Gene Expression Profiling
Source: Cancers (Basel). 2023 Nov 4;15(21):5289. doi: 10.3390/cancers15215289 (PMC10648390; doi:10.3390/cancers15215289)
Supplement: Supplementary file 1 [file cancers-15-05289-s001.zip › Table S2.pdf]

Table S2. List of 404 differentially expressed genes.

Gene	Entrezgene	Name	HGNC
ABCB9	23457	ATP binding cassette subfamily B member 9	HGNC:50
ABCG1	9619	ATP binding cassette subfamily G member 1	HGNC:73
ABHD15	116236	abhydrolase domain containing 15	HGNC:26971
ABHD3	171586	abhydrolase domain containing 3, phospholipase	HGNC:18718
ABLIM1	3983	actin binding LIM protein 1	HGNC:78
ACOT7	11332	acyl-CoA thioesterase 7	HGNC:24157
ACTA2	59	actin alpha 2, smooth muscle	HGNC:130
ADCY2	108	adenylate cyclase 2	HGNC:233
ADPRH	141	ADP-ribosylarginine hydrolase	HGNC:269
ADRB2	154	adrenoceptor beta 2	HGNC:286
AIFM3	150209	apoptosis inducing factor mitochondria associated 3	HGNC:26398
AK1	203	adenylate kinase 1	HGNC:361
ALDH1L2	160428	aldehyde dehydrogenase 1 family member L2	HGNC:26777
ANKRD36BP2	645784	ankyrin repeat domain 36B pseudogene 2	HGNC:33607
ANO9	338440	anoctamin 9	HGNC:20679
AQP1	358	aquaporin 1 (Colton blood group)	HGNC:633
ARHGEF10	9639	Rho guanine nucleotide exchange factor 10	HGNC:14103
ARHGEF40	55701	Rho guanine nucleotide exchange factor 40	HGNC:25516
ARSD	414	arylsulfatase D	HGNC:717
ARTN	9048	artemin	HGNC:727
ARVCF	421	ARVCF delta catenin family member	HGNC:728
ATF7IP2	80063	activating transcription factor 7 interacting protein 2	HGNC:20397
ATG12	9140	autophagy related 12	HGNC:588
ATXN7L1	222255	ataxin 7 like 1	HGNC:22210
AURKA	6790	aurora kinase A	HGNC:11393

BACE2	25825	beta-secretase 2	HGNC:934
BBS2	583	Bardet-Biedl syndrome 2	HGNC:967
BBS9	27241	Bardet-Biedl syndrome 9	HGNC:30000
BIN1	274	bridging integrator 1	HGNC:1052
BIRC5	332	baculoviral IAP repeat containing 5	HGNC:593
BTF3	689	basic transcription factor 3	HGNC:1125
BUB1	699	BUB1 mitotic checkpoint serine/threonine kinase	HGNC:1148
C16orf86	388284	chromosome 16 open reading frame 86	HGNC:33755
C5orf15	56951	chromosome 5 open reading frame 15	HGNC:20656
CACNA2D4	93589	calcium voltage-gated channel auxiliary subunit alpha2delta 4	HGNC:20202
CALN1	83698	calneuron 1	HGNC:13248
CAMLG	819	calcium modulating ligand	HGNC:1471
CARD9	64170	caspase recruitment domain family member 9	HGNC:16391
CATSPER1	117144	cation channel sperm associated 1	HGNC:17116
CCDC106	29903	coiled-coil domain containing 106	HGNC:30181
CCDC15	80071	coiled-coil domain containing 15	HGNC:25798
CCDC85C	317762	coiled-coil domain containing 85C	HGNC:35459
CCL23	6368	C-C motif chemokine ligand 23	HGNC:10622
CCT6B	10693	chaperonin containing TCP1 subunit 6B	HGNC:1621
CD320	51293	CD320 molecule	HGNC:16692
CD82	3732	CD82 molecule	HGNC:6210
CDC20	991	cell division cycle 20	HGNC:1723
CDC42BPA	8476	CDC42 binding protein kinase alpha	HGNC:1737
CDCA2	157313	cell division cycle associated 2	HGNC:14623
CDCA5	113130	cell division cycle associated 5	HGNC:14626
CDCA8	55143	cell division cycle associated 8	HGNC:14629
CDH1	999	cadherin 1	HGNC:1748
CFD	1675	complement factor D	HGNC:2771
CHAF1B	8208	chromatin assembly factor 1 subunit B	HGNC:1911

CHEK1	1111	checkpoint kinase 1	HGNC:1925
CHI3L1	1116	chitinase 3 like 1	HGNC:1932
CKAP2L	150468	cytoskeleton associated protein 2 like	HGNC:26877
CKAP5	9793	cytoskeleton associated protein 5	HGNC:28959
CKB	1152	creatine kinase B	HGNC:1991
CLCN4	1183	chloride voltage-gated channel 4	HGNC:2022
CLIP3	25999	CAP-Gly domain containing linker protein 3	HGNC:24314
CNOT8	9337	CCR4-NOT transcription complex subunit 8	HGNC:9207
COL18A1	80781	collagen type XVIII alpha 1 chain	HGNC:2195
COL9A2	1298	collagen type IX alpha 2 chain	HGNC:2218
COMMD10	51397	COMM domain containing 10	HGNC:30201
COMMD6	170622	COMM domain containing 6	HGNC:24015
COPG2	26958	COPI coat complex subunit gamma 2	HGNC:2237
COX4I1	1327	cytochrome c oxidase subunit 4I1	HGNC:2265
CRYBG3	131544	crystallin beta-gamma domain containing 3	HGNC:34427
CSF1	1435	colony stimulating factor 1	HGNC:2432
CSNK1G3	1456	casein kinase 1 gamma 3	HGNC:2456
CTSO	1519	cathepsin O	HGNC:2542
CYB5R3	1727	cytochrome b5 reductase 3	HGNC:2873
CYSLTR2	57105	cysteinyl leukotriene receptor 2	HGNC:18274
DAAM1	23002	dishevelled associated activator of morphogenesis 1	HGNC:18142
DEPDC1	55635	DEP domain containing 1	HGNC:22949
DERA	51071	deoxyribose-phosphate aldolase	HGNC:24269
DGAT1	8694	diacylglycerol O-acyltransferase 1	HGNC:2843
DHCR7	1717	7-dehydrocholesterol reductase	HGNC:2860
DHRS13	147015	dehydrogenase/reductase 13	HGNC:28326
DMXL2	23312	Dmx like 2	HGNC:2938
DNAJC9	23234	DnaJ heat shock protein family (Hsp40) member C9	HGNC:19123
DPEP2	64174	dipeptidase 2	HGNC:23028

DSC2	1824	desmocollin 2	HGNC:3036
DTWD2	285605	DTW domain containing 2	HGNC:19334
E2F8	79733	E2F transcription factor 8	HGNC:24727
EFCAB2	84288	EF-hand calcium binding domain 2	HGNC:28166
EFHC1	114327	EF-hand domain containing 1	HGNC:16406
ELOVL6	79071	ELOVL fatty acid elongase 6	HGNC:15829
EMILIN1	11117	elastin microfibril interfacer 1	HGNC:19880
EMP1	2012	epithelial membrane protein 1	HGNC:3333
ENDOD1	23052	endonuclease domain containing 1	HGNC:29129
ERAP1	51752	endoplasmic reticulum aminopeptidase 1	HGNC:18173
ESPL1	9700	extra spindle pole bodies like 1, separase	HGNC:16856
EVI2A	2123	ecotropic viral integration site 2A	HGNC:3499
FAM110B	90362	family with sequence similarity 110 member B	HGNC:28587
FAM13B	51306	family with sequence similarity 13 member B	HGNC:1335
FAM83D	81610	family with sequence similarity 83 member D	HGNC:16122
FAM89A	375061	family with sequence similarity 89 member A	HGNC:25057
FARP1	10160	FERM, ARH/RhoGEF and pleckstrin domain protein 1	HGNC:3591
FBXL17	64839	F-box and leucine rich repeat protein 17	HGNC:13615
FBXO38	81545	F-box protein 38	HGNC:28844
FGD5	152273	FYVE, RhoGEF and PH domain containing 5	HGNC:19117
FHL2	2274	four and a half LIM domains 2	HGNC:3703
FHL3	2275	four and a half LIM domains 3	HGNC:3704
FKBP4	2288	FKBP prolyl isomerase 4	HGNC:3720
FN3K	64122	fructosamine 3 kinase	HGNC:24822
FOXM1	2305	forkhead box M1	HGNC:3818
FOXRED2	80020	FAD dependent oxidoreductase domain containing 2	HGNC:26264
FSD1L	83856	fibronectin type III and SPRY domain containing 1 like	HGNC:13753
FTO	79068	FTO alpha-ketoglutarate dependent dioxygenase	HGNC:24678
FUT8	2530	fucosyltransferase 8	HGNC:4019

GALE	2582	UDP-galactose-4-epimerase	HGNC:4116
GALNT11	63917	polypeptide N-acetylgalactosaminyltransferase 11	HGNC:19875
GAPT	202309	GRB2 binding adaptor protein, transmembrane	HGNC:26588
GAS2L1	10634	growth arrest specific 2 like 1	HGNC:16955
GATA1	2623	GATA binding protein 1	HGNC:4170
GATAD1	57798	GATA zinc finger domain containing 1	HGNC:29941
GCLM	2730	glutamate-cysteine ligase modifier subunit	HGNC:4312
GFI1B	8328	growth factor independent 1B transcriptional repressor	HGNC:4238
GIPC1	10755	GIPC PDZ domain containing family member 1	HGNC:1226
GM2A	2760	ganglioside GM2 activator	HGNC:4367
GRAP2	9402	GRB2 related adaptor protein 2	HGNC:4563
GSTK1	373156	glutathione S-transferase kappa 1	HGNC:16906
GTF2IRD2	84163	GTF2I repeat domain containing 2	HGNC:30775
GTF2IRD2B	389524	GTF2I repeat domain containing 2B	HGNC:33125
GTSE1	51512	G2 and S-phase expressed 1	HGNC:13698
HARS2	23438	histidyl-tRNA synthetase 2, mitochondrial	HGNC:4817
HAUS8	93323	HAUS augmin like complex subunit 8	HGNC:30532
HAVCR2	84868	hepatitis A virus cellular receptor 2	HGNC:18437
HBG1	3047	hemoglobin subunit gamma 1	HGNC:4831
HBG2	3048	hemoglobin subunit gamma 2	HGNC:4832
HHAT	55733	hedgehog acyltransferase	HGNC:18270
HIGD2A	192286	HIG1 hypoxia inducible domain family member 2A	HGNC:28311
HINT1	3094	histidine triad nucleotide binding protein 1	HGNC:4912
HJURP	55355	Holliday junction recognition protein	HGNC:25444
HMBS	3145	hydroxymethylbilane synthase	HGNC:4982
HMGN5	79366	high mobility group nucleosome binding domain 5	HGNC:8013
HNMT	3176	histamine N-methyltransferase	HGNC:5028
HOXA11	3207	homeobox A11	HGNC:5101
HOXA3	3200	homeobox A3	HGNC:5104

HOXA4	3201	homeobox A4	HGNC:5105
HOXA6	3203	homeobox A6	HGNC:5107
HOXB3	3213	homeobox B3	HGNC:5114
HOXB4	3214	homeobox B4	HGNC:5115
HOXB6	3216	homeobox B6	HGNC:5117
HOXB7	3217	homeobox B7	HGNC:5118
HOXB8	3218	homeobox B8	HGNC:5119
HOXB9	3219	homeobox B9	HGNC:5120
HSD17B4	3295	hydroxysteroid 17-beta dehydrogenase 4	HGNC:5213
HSPB1	3315	heat shock protein family B (small) member 1	HGNC:5246
HSPG2	3339	heparan sulfate proteoglycan 2	HGNC:5273
ICAM4	3386	intercellular adhesion molecule 4 (Landsteiner-Wiener blood grou[illegible]	HGNC:5347
IFITM1	8519	interferon induced transmembrane protein 1	HGNC:5412
IFITM2	10581	interferon induced transmembrane protein 2	HGNC:5413
IGSF10	285313	immunoglobulin superfamily member 10	HGNC:26384
IL12A	3592	interleukin 12A	HGNC:5969
IL7	3574	interleukin 7	HGNC:6023
ILDR2	387597	immunoglobulin like domain containing receptor 2	HGNC:18131
IMMP2L	83943	inner mitochondrial membrane peptidase subunit 2	HGNC:14598
ING3	54556	inhibitor of growth family member 3	HGNC:14587
IQSEC2	23096	IQ motif and Sec7 domain ArfGEF 2	HGNC:29059
IRX3	79191	iroquois homeobox 3	HGNC:14360
IRX5	10265	iroquois homeobox 5	HGNC:14361
ITGAE	3682	integrin subunit alpha E	HGNC:6147
ITPR3	3710	inositol 1,4,5-trisphosphate receptor type 3	HGNC:6182
ITPRIPL1	150771	ITPRIP like 1	HGNC:29371
JAM3	83700	junctional adhesion molecule 3	HGNC:15532
KANK2	25959	KN motif and ankyrin repeat domains 2	HGNC:29300
KCTD1	284252	potassium channel tetramerization domain containing 1	HGNC:18249

KCTD9	54793	potassium channel tetramerization domain containing 9	HGNC:22401
KIAA1549	57670	KIAA1549	HGNC:22219
KIF26B	55083	kinesin family member 26B	HGNC:25484
KIF27	55582	kinesin family member 27	HGNC:18632
KIF2C	11004	kinesin family member 2C	HGNC:6393
KIF3C	3797	kinesin family member 3C	HGNC:6321
KIF4A	24137	kinesin family member 4A	HGNC:13339
KLHDC8B	200942	kelch domain containing 8B	HGNC:28557
KLRG1	10219	killer cell lectin like receptor G1	HGNC:6380
LIPE	3991	lipase E, hormone sensitive type	HGNC:6621
LNPEP	4012	leucyl and cystinyl aminopeptidase	HGNC:6656
LRBA	987	LPS responsive beige-like anchor protein	HGNC:1742
LRFN4	78999	leucine rich repeat and fibronectin type III domain containing 4	HGNC:28456
LRRC23	10233	leucine rich repeat containing 23	HGNC:19138
LRRC27	80313	leucine rich repeat containing 27	HGNC:29346
LRRN2	10446	leucine rich repeat neuronal 2	HGNC:16914
LSR	51599	lipolysis stimulated lipoprotein receptor	HGNC:29572
LTK	4058	leukocyte receptor tyrosine kinase	HGNC:6721
LY6E	4061	lymphocyte antigen 6 family member E	HGNC:6727
MAP3K1	4214	mitogen-activated protein kinase kinase kinase 1	HGNC:6848
MED7	9443	mediator complex subunit 7	HGNC:2378
MFHAS1	9258	multifunctional ROCO family signaling regulator 1	HGNC:16982
MFSD1	64747	major facilitator superfamily domain containing 1	HGNC:25874
MFSD2B	388931	MFSD2 lysolipid transporter B, sphingolipid	HGNC:37207
MLYCD	23417	malonyl-CoA decarboxylase	HGNC:7150
MMD	23531	monocyte to macrophage differentiation associated	HGNC:7153
MRPS33	51650	mitochondrial ribosomal protein S33	HGNC:16634
MYCT1	80177	MYC target 1	HGNC:23172
MYH10	4628	myosin heavy chain 10	HGNC:7568

MYL4	4635	myosin light chain 4	HGNC:7585
NAGPA	51172	N-acetylglucosamine-1-phosphodiester alpha-N-acetylglucosamini	HGNC:17378
NAV1	89796	neuron navigator 1	HGNC:15989
NBR2	10230	neighbor of BRCA1 lncRNA 2	HGNC:20691
NCAPD3	23310	non-SMC condensin II complex subunit D3	HGNC:28952
NCAPH	23397	non-SMC condensin I complex subunit H	HGNC:1112
NCKAP1	10787	NCK associated protein 1	HGNC:7666
NDUFB5	4711	NADH:ubiquinone oxidoreductase subunit B5	HGNC:7700
NEDD4L	23327	NEDD4 like E3 ubiquitin protein ligase	HGNC:7728
NEK10	152110	NIMA related kinase 10	HGNC:18592
NEK2	4751	NIMA related kinase 2	HGNC:7745
NPW	283869	neuropeptide W	HGNC:30509
NQO1	1728	NAD(P)H quinone dehydrogenase 1	HGNC:2874
NSA2	10412	NSA2 ribosome biogenesis factor	HGNC:30728
NUDT7	283927	nudix hydrolase 7	HGNC:8054
NUF2	83540	NUF2 component of NDC80 kinetochore complex	HGNC:14621
OAF	220323	out at first homolog	HGNC:28752
OPTN	10133	optineurin	HGNC:17142
ORAI2	80228	ORAI calcium release-activated calcium modulator 2	HGNC:21667
ORC1	4998	origin recognition complex subunit 1	HGNC:8487
OTUD7B	56957	OTU deubiquitinase 7B	HGNC:16683
PAFAH1B3	5050	platelet activating factor acetylhydrolase 1b catalytic subunit 3	HGNC:8576
PARVB	29780	parvin beta	HGNC:14653
PBX3	5090	PBX homeobox 3	HGNC:8634
PC	5091	pyruvate carboxylase	HGNC:8636
PCBD2	84105	pterin-4 alpha-carbinolamine dehydratase 2	HGNC:24474
PCYT1B	9468	phosphate cytidylyltransferase 1B, choline	HGNC:8755
PGGT1B	5229	protein geranylgeranyltransferase type I subunit beta	HGNC:8895
PI15	51050	peptidase inhibitor 15	HGNC:8946

PIK3CG	5294	phosphatidylinositol-4,5-bisphosphate 3-kinase catalytic subunit g	HGNC:8978
PKLR	5313	pyruvate kinase L/R	HGNC:9020
PKN3	29941	protein kinase N3	HGNC:17999
PLCB4	5332	phospholipase C beta 4	HGNC:9059
PLEKHG4	25894	pleckstrin homology and RhoGEF domain containing G4	HGNC:24501
PMPCB	9512	peptidase, mitochondrial processing subunit beta	HGNC:9119
PNMA1	9240	PNMA family member 1	HGNC:9158
POLN	353497	DNA polymerase nu	HGNC:18870
POU2AF1	5450	POU class 2 homeobox associating factor 1	HGNC:9211
PPAN	56342	peter pan homolog	HGNC:9227
PRDX4	10549	peroxiredoxin 4	HGNC:17169
PROS1	5627	protein S	HGNC:9456
PRR5	55615	proline rich 5	HGNC:31682
PRRT3	285368	proline rich transmembrane protein 3	HGNC:26591
PRX	57716	periaxin	HGNC:13797
PSMB10	5699	proteasome 20S subunit beta 10	HGNC:9538
PSMG1	8624	proteasome assembly chaperone 1	HGNC:3043
PTGDS	5730	prostaglandin D2 synthase	HGNC:9592
PYCARD	29108	PYD and CARD domain containing	HGNC:16608
PYCR1	5831	pyrroline-5-carboxylate reductase 1	HGNC:9721
RAB30	27314	RAB30, member RAS oncogene family	HGNC:9770
RAB33A	9363	RAB33A, member RAS oncogene family	HGNC:9773
RAB3IL1	5866	RAB3A interacting protein like 1	HGNC:9780
RAC3	5881	Rac family small GTPase 3	HGNC:9803
RAD54L	8438	RAD54 like	HGNC:9826
REC8	9985	REC8 meiotic recombination protein	HGNC:16879
RECQL4	9401	RecQ like helicase 4	HGNC:9949
REXO2	25996	RNA exonuclease 2	HGNC:17851
RIMS3	9783	regulating synaptic membrane exocytosis 3	HGNC:21292

RNF135	84282	ring finger protein 135	HGNC:21158
RPGR	6103	retinitis pigmentosa GTPase regulator	HGNC:10295
RPL12	6136	ribosomal protein L12	HGNC:10302
RPL15	6138	ribosomal protein L15	HGNC:10306
RPL17	6139	ribosomal protein L17	HGNC:10307
RPL22L1	200916	ribosomal protein L22 like 1	HGNC:27610
RPL23	9349	ribosomal protein L23	HGNC:10316
RPL26	6154	ribosomal protein L26	HGNC:10327
RPL27	6155	ribosomal protein L27	HGNC:10328
RPL34	6164	ribosomal protein L34	HGNC:10340
RPL35A	6165	ribosomal protein L35a	HGNC:10345
RPL38	6169	ribosomal protein L38	HGNC:10349
RPL39	6170	ribosomal protein L39	HGNC:10350
RPS10	6204	ribosomal protein S10	HGNC:10383
RPS14	6208	ribosomal protein S14	HGNC:10387
RPS23	6228	ribosomal protein S23	HGNC:10410
RPS27	6232	ribosomal protein S27	HGNC:10416
RPS27L	51065	ribosomal protein S27 like	HGNC:18476
RPS4X	6191	ribosomal protein S4 X-linked	HGNC:10424
RRN3P2	653390	RRN3 pseudogene 2	HGNC:37619
RRP1B	23076	ribosomal RNA processing 1B	HGNC:23818
RYR3	6263	ryanodine receptor 3	HGNC:10485
S100B	6285	S100 calcium binding protein B	HGNC:10500
SAGE1	55511	sarcoma antigen 1	HGNC:30369
SCD	6319	stearoyl-CoA desaturase	HGNC:10571
SCUBE1	80274	signal peptide, CUB domain and EGF like domain containing 1	HGNC:13441
SEC11A	23478	SEC11 homolog A, signal peptidase complex subunit	HGNC:17718
SEC14L2	23541	SEC14 like lipid binding 2	HGNC:10699
SEMA3C	10512	semaphorin 3C	HGNC:10725

SEMA4B	10509	semaphorin 4B	HGNC:10730
SERPINE2	5270	serpin family E member 2	HGNC:8951
SERPINI1	5274	serpin family I member 1	HGNC:8943
SGMS1	259230	sphingomyelin synthase 1	HGNC:29799
SGPP1	81537	sphingosine-1-phosphate phosphatase 1	HGNC:17720
SH2D3A	10045	SH2 domain containing 3A	HGNC:16885
SH3BGRL2	83699	SH3 domain binding glutamate rich protein like 2	HGNC:15567
SH3BP5	9467	SH3 domain binding protein 5	HGNC:10827
SIGMAR1	10280	sigma non-opioid intracellular receptor 1	HGNC:8157
SIL1	64374	SIL1 nucleotide exchange factor	HGNC:24624
SKA1	220134	spindle and kinetochore associated complex subunit 1	HGNC:28109
SLC18A2	6571	solute carrier family 18 member A2	HGNC:10935
SLC1A5	6510	solute carrier family 1 member 5	HGNC:10943
SLC22A23	63027	solute carrier family 22 member 23	HGNC:21106
SLC25A23	79085	solute carrier family 25 member 23	HGNC:19375
SLC25A35	399512	solute carrier family 25 member 35	HGNC:31921
SLC26A2	1836	solute carrier family 26 member 2	HGNC:10994
SLC29A2	3177	solute carrier family 29 member 2	HGNC:11004
SLC2A1	6513	solute carrier family 2 member 1	HGNC:11005
SLC2A13	114134	solute carrier family 2 member 13	HGNC:15956
SLC2A9	56606	solute carrier family 2 member 9	HGNC:13446
SLC38A5	92745	solute carrier family 38 member 5	HGNC:18070
SLC41A1	254428	solute carrier family 41 member 1	HGNC:19429
SLC43A1	8501	solute carrier family 43 member 1	HGNC:9225
SLC43A3	29015	solute carrier family 43 member 3	HGNC:17466
SLC44A2	57153	solute carrier family 44 member 2	HGNC:17292
SLC7A1	6541	solute carrier family 7 member 1	HGNC:11057
SLCO4A1	28231	solute carrier organic anion transporter family member 4A1	HGNC:10953
SMAD5	4090	SMAD family member 5	HGNC:6771

SMAGP	57228	small cell adhesion glycoprotein	HGNC:26918
SNHG5	387066	small nucleolar RNA host gene 5	HGNC:21026
SNPH	9751	syntaphilin	HGNC:15931
SNX2	6643	sorting nexin 2	HGNC:11173
SOD1	6647	superoxide dismutase 1	HGNC:11179
SORBS1	10580	sorbin and SH3 domain containing 1	HGNC:14565
SPINT2	10653	serine peptidase inhibitor, Kunitz type 2	HGNC:11247
SPMIP4	136895	sperm microtubule inner protein 4	HGNC:21722
SPTBN2	6712	spectrin beta, non-erythrocytic 2	HGNC:11276
SQLE	6713	squalene epoxidase	HGNC:11279
SRC	6714	SRC proto-oncogene, non-receptor tyrosine kinase	HGNC:11283
SREBF2	6721	sterol regulatory element binding transcription factor 2	HGNC:11290
SSBP1	6742	single stranded DNA binding protein 1	HGNC:11317
SSBP3	23648	single stranded DNA binding protein 3	HGNC:15674
ST8SIA4	7903	ST8 alpha-N-acetyl-neuraminide alpha-2,8-sialyltransferase 4	HGNC:10871
STAC3	246329	SH3 and cysteine rich domain 3	HGNC:28423
STARD10	10809	StAR related lipid transfer domain containing 10	HGNC:10666
STIL	6491	STIL centriolar assembly protein	HGNC:10879
STS	412	steroid sulfatase	HGNC:11425
SV2B	9899	synaptic vesicle glycoprotein 2B	HGNC:16874
SYNGAP1	8831	synaptic Ras GTPase activating protein 1	HGNC:11497
SYNJ2	8871	synaptojanin 2	HGNC:11504
TAL1	6886	TAL bHLH transcription factor 1, erythroid differentiation factor	HGNC:11556
TAX1BP1	8887	Tax1 binding protein 1	HGNC:11575
TBC1D4	9882	TBC1 domain family member 4	HGNC:19165
TBPL1	9519	TATA-box binding protein like 1	HGNC:11589
TBXAS1	6916	thromboxane A synthase 1	HGNC:11609
TC2N	123036	tandem C2 domains, nuclear	HGNC:19859
TCTN1	79600	tectonic family member 1	HGNC:26113

TDRKH	11022	tudor and KH domain containing	HGNC:11713
TFDP1	7027	transcription factor Dp-1	HGNC:11749
TFEC	22797	transcription factor EC	HGNC:11754
TGFB1I1	7041	transforming growth factor beta 1 induced transcript 1	HGNC:11767
TGM2	7052	transglutaminase 2	HGNC:11778
TGS1	96764	trimethylguanosine synthase 1	HGNC:17843
THG1L	54974	tRNA-histidine guanylyltransferase 1 like	HGNC:26053
THSD7A	221981	thrombospondin type 1 domain containing 7A	HGNC:22207
TIAM1	7074	TIAM Rac1 associated GEF 1	HGNC:11805
TIGD6	81789	tigger transposable element derived 6	HGNC:18332
TIMP3	7078	TIMP metallopeptidase inhibitor 3	HGNC:11822
TK2	7084	thymidine kinase 2	HGNC:11831
TMCO4	255104	transmembrane and coiled-coil domains 4	HGNC:27393
TMED7	51014	transmembrane p24 trafficking protein 7	HGNC:24253
TMEM105	284186	TMEM105 long non-coding RNA	HGNC:26794
TMEM106A	113277	transmembrane protein 106A	HGNC:28288
TMEM63B	55362	transmembrane protein 63B	HGNC:17735
TMT1A	25840	thiol methyltransferase 1A	HGNC:24550
TNFAIP8	25816	TNF alpha induced protein 8	HGNC:17260
TNFRSF25	8718	TNF receptor superfamily member 25	HGNC:11910
TNFSF12	8742	TNF superfamily member 12	HGNC:11927
TNFSF13	8741	TNF superfamily member 13	HGNC:11928
TNXB	7148	tenascin XB	HGNC:11976
TOMM7	54543	translocase of outer mitochondrial membrane 7	HGNC:21648
TPK1	27010	thiamin pyrophosphokinase 1	HGNC:17358
TPM1	7168	tropomyosin 1	HGNC:12010
TPST2	8459	tyrosylprotein sulfotransferase 2	HGNC:12021
TPX2	22974	TPX2 microtubule nucleation factor	HGNC:1249
TRIB2	28951	tribbles pseudokinase 2	HGNC:30809

TRIP10	9322	thyroid hormone receptor interactor 10	HGNC:12304
TROAP	10024	trophinin associated protein	HGNC:12327
TSPAN31	6302	tetraspanin 31	HGNC:10539
TSPAN5	10098	tetraspanin 5	HGNC:17753
TTC39B	158219	tetratricopeptide repeat domain 39B	HGNC:23704
TUBG1	7283	tubulin gamma 1	HGNC:12417
UBASH3A	53347	ubiquitin associated and SH3 domain containing A	HGNC:12462
UBXN10	127733	UBX domain protein 10	HGNC:26354
UROD	7389	uroporphyrinogen decarboxylase	HGNC:12591
UROS	7390	uroporphyrinogen III synthase	HGNC:12592
USP44	84101	ubiquitin specific peptidase 44	HGNC:20064
VIM	7431	vimentin	HGNC:12692
WDR5B	54554	WD repeat domain 5B	HGNC:17826
WDR62	284403	WD repeat domain 62	HGNC:24502
WEE1	7465	WEE1 G2 checkpoint kinase	HGNC:12761
XRCC4	7518	X-ray repair cross complementing 4	HGNC:12831
YWHAH	7533	tyrosine 3-monooxygenase/tryptophan 5-monooxygenase activatio	HGNC:12853
ZCWPW1	55063	zinc finger CW-type and PWWP domain containing 1	HGNC:23486
ZDHHC21	340481	zinc finger DHHC-type palmitoyltransferase 21	HGNC:20750
ZIK1	284307	zinc finger protein interacting with K protein 1	HGNC:33104
ZNF208	7757	zinc finger protein 208	HGNC:12999
ZNF229	7772	zinc finger protein 229	HGNC:13022
ZNF277	11179	zinc finger protein 277	HGNC:13070
ZNF347	84671	zinc finger protein 347	HGNC:16447
ZNF354C	30832	zinc finger protein 354C	HGNC:16736
ZNF514	84874	zinc finger protein 514	HGNC:25894
ZNF528	84436	zinc finger protein 528	HGNC:29384
ZNF585B	92285	zinc finger protein 585B	HGNC:30948
ZNF804A	91752	zinc finger protein 804A	HGNC:21711

ZNF90	7643	zinc finger protein 90	HGNC:13165
ZSWIM7	125150	zinc finger SWIM-type containing 7	HGNC:26993

HGNC The HUGO Gene Nomenclature Committee.
