# Supplementary material for: Identification of a Complex Karyotype Signature with Clinical Implications in AML and MDS-EB Using Gene Expression Profiling
Source: Cancers (Basel). 2023 Nov 4;15(21):5289. doi: 10.3390/cancers15215289 (PMC10648390; doi:10.3390/cancers15215289)
Supplement: Supplementary file 1 [file cancers-15-05289-s001.zip › Table S3.pdf]

Table S3. Pathways identified in overrepresentation anlaysis.

Pathway	Set size	Candidates Contained	*p* -value	*q* -value	Pathway source	Members Input Overlap
Nonsense-Mediated Decay (NMD)	117	17 (14.5%)	<0.0001	<0.0001	Reactome	*RPL22L1; RPS27; RPS10; RPS23; RPS27L; RPL39; RPL27; RPL26; RPL35A; RPL23; RPL15; RPL17; RPS14; RPL12; RPS4X; RPL38; RPL34*
Cytoplasmic Ribosomal Proteins	88	15 (17.0%)	<0.0001	<0.0001	Wikipathways	*RPS27; RPS10; RPS23; RPL38; RPL39; RPL34; RPL26; RPL35A; RPL23; RPL15; RPL17; RPS14; RPL12; RPS4X; RPL27*
Ribosome - Homo sapiens (human)	158	17 (10.8%)	<0.0001	<0.0001	KEGG	*RPL22L1; RPS27; RPS10; RPS23; RPL38; RPL39; RPL27; RPL26; RPL35A; RPL23; RPL15; RPL17; RPS14; RPL12; RPS4X; RPL34; RPS27L*
Translation	307	20 (6.5%)	<0.0001	0.0007	Reactome	*RPL22L1; RPS27; RPS10; SEC11A; RPS23; RPS27L; RPL39; RPL27; RPL26; RPL35A; RPL23; RPL15; RPL17; RPS14; MRPS33; RPL12; RPS4X; RPL38; RPL34; HARS2*
Metabolism of amino acids and derivatives	339	21 (6.2%)	<0.0001	0.0008	Reactome	*RPL22L1; RPS27; HNMT; RPS10; RPS23; RPS27L; RPL39; RPL27; RPL26; RPL35A; RPL23; NQO1; RPL15; RPL17; RPS14; RPL12; RPS4X; RPL38; PYCR1; CKB; RPL34*
Mitotic Prometaphase	192	14 (7.3%)	0.0001	0.0031	Reactome	*RPS27; NEK2; TUBG1; HAUS8; NUF2; BIRC5; CDCA5; CDC20; SKA1; NCAPH; BUB1; CDCA8; KIF2C; CKAP5*
Regulation of cholesterol biosynthesis by SREBP (SREBF)	31	5 (16.1%)	0.0006	0.0112	Reactome	*ELOVL6; SCD; SREBF2; SQLE; DHCR7*
Integrin cell surface interactions	66	7 (10.6%)	0.0007	0.0125	Reactome	*JAM3; COL18A1; HSPG2; COL9A2; ICAM4; CDH1; ITGAE*
Heme Biosynthesis	9	3 (33.3%)	0.0008	0.0153	Wikipathways	*UROD; HMBS; UROS*
Mitotic Spindle Checkpoint	114	9 (7.9%)	0.0010	0.0182	Reactome	*RPS27; BUB1; NUF2; BIRC5; CDC20; SKA1; CDCA8; KIF2C; CKAP5*
G2/M Transition	138	10 (7.2%)	0.0011	0.0184	Reactome	*TPX2; OPTN; NEK2; HAUS8; GTSE1; TUBG1; FOXM1; AURKA; WEE1; CKAP5*
Signaling by Rho GTPases, Miro GTPases and RHOBTB3	705	29 (4.1%)	0.0011	0.0186	Reactome	*MYH10; FGD5; CKB; BIRC5; FAM13B; CDC20; KIF2C; FARP1; SKA1; TIAM1; YWHAH; CDC42BPA; CDH1; CDCA8; NCKAP1; ARHGEF10; RPS27; BUB1; RAC3; NUF2; DAAM1; PKN3; SRC; TRIP10; PLEKHG4; VIM; SLC1A5; CKAP5; ARHGEF40*
Cellular responses to stress	554	24 (4.3%)	0.0015	0.0221	Reactome	*RPS10; RPS27L; RPS14; RPL35A; FKBP4; RPL22L1; RPL27; RPL26; RPL23; FBXL17; RPS27; RPS23; HSPB1; SOD1; RPL15; COX4I1; TGS1; RPL12; RPS4X; RPL38; RPL39; RPL34; RPL17; TFDP1*
Gamma carboxylation, hypusine formation and arylsulfatase activation	41	5 (12.2%)	0.0021	0.0291	Reactome	*ARSD; PROS1; FN3K; TPST2; STS*
Kinesins	43	5 (11.6%)	0.0026	0.0345	Reactome	*KIF27; KIF26B; KIF4A; KIF2C; KIF3C*
Biosynthesis of unsaturated fatty acids - Homo sapiens (human)	27	4 (14.8%)	0.0029	0.0362	KEGG	*ELOVL6; SCD; HSD17B4; ACOT7*
SLC-mediated transmembrane transport	243	13 (5.3%)	0.0032	0.0405	Reactome	*SLC41A1; SLC38A5; SLC44A2; SLC2A9; SLC2A13; SLC2A1; SLC7A1; SLC26A2; SLC29A2; SLC1A5; SLC18A2; SLCO4A1; SLC43A1*
Heme biosynthesis	14	3 (21.4%)	0.0033	0.0408	Reactome	*UROD; HMBS; UROS*
Cholesterol metabolism (includes both Bloch and Kandutsch-Russell pathways)	46	5 (10.9%)	0.0035	0.0408	Wikipathways	*SREBF2; SCD; ABCG1; SQLE; DHCR7*
Apelin signaling pathway - Homo sapiens (human)	137	9 (6.6%)	0.0036	0.0414	KEGG	*ACTA2; PLCB4; ADCY2; RYR3; PIK3CG; LIPE; MYL4; CDH1; ITPR3*
Synthesis of PC	29	4 (13.8%)	0.0037	0.0414	Reactome	*STARD10; ABHD3; SLC44A2; PCYT1B*
Regulation of sister chromatid separation at the metaphase-anaphase transition	15	3 (20.0%)	0.0041	0.0446	Wikipathways	*ESPL1; BUB1; CDC20*
Mitotic Anaphase	195	11 (5.6%)	0.0044	0.0470	Reactome	*RPS27; BUB1; NUF2; BIRC5; CDC20; SKA1; ESPL1; CDCA5; CDCA8; KIF2C; CKAP5*
Golgi-to-ER retrograde transport	116	8 (6.9%)	0.0045	0.0470	Reactome	*TMED7; KIF27; KIF26B; COPG2; PAFAH1B3; KIF4A; KIF3C; KIF2C*
Cell cycle	120	8 (6.7%)	0.0055	0.0546	Wikipathways	*BUB1; CDC20; YWHAH; ESPL1; WEE1; ORC1; TFDP1; CHEK1*
Cell cycle - Homo sapiens	124	8 (6.5%)	0.0066	0.0646	KEGG	*BUB1; ORC1; YWHAH; ESPL1; WEE1; CDC20; TFDP1; CHEK1*
Oocyte meiosis - Homo sapiens	129	8 (6.2%)	0.0084	0.0792	KEGG	*BUB1; ADCY2; YWHAH; CDC20; REC8; ESPL1; AURKA; ITPR3*

KEGG The Kyoto Encyclopedia of Genes and Genomes.
