# Supplementary material for: Identification of a Complex Karyotype Signature with Clinical Implications in AML and MDS-EB Using Gene Expression Profiling
Source: Cancers (Basel). 2023 Nov 4;15(21):5289. doi: 10.3390/cancers15215289 (PMC10648390; doi:10.3390/cancers15215289)
Supplement: Supplementary file 1 [file cancers-15-05289-s001.zip › Table S4.pdf]

Table S4. List of discrepant cases in the prediction of CKS of karyotype with karyotype and microarray information.

ID	Cohort	Diagnosis	Karyotype	Microarray	Karyotype group	CKS
aml_ohsu_2018_14_00152	BeatAML	AML	46,XY,add(8)(p21),t(8;19)(p11.2;q13.3)[18]/46,XY[2]	NA	Non-complex	CKS
aml_ohsu_2018_14_00774	BeatAML	AML	46,XY[20]	NA	Non-complex	CKS
aml_ohsu_2018_16_00145	BeatAML	AML	46,XY,t(1;12)(p32;p13)[3]/46,XY[17]	NA	Non-complex	CKS
TCGA-AB-2882	TCGA	AML	45,XX,-7[12]/46,XX[8]	Non-complex	Non-complex	CKS
TCGA-AB-2814	TCGA	AML	46,XX,del(5)(q22q33)[4]/46,XX[16]	Non-complex	Non-complex	CKS
TCGA-AB-2938	TCGA	AML	45,X,-Y[3]/46,XY [17]	Complex	Non-complex	CKS
TCGA-AB-2987	TCGA	AML	46,XX[18]	Non-complex	Non-complex	CKS
K32	KUMC	AML	46,XY,dup(1)(q23q32)[2]/46,XY[18]	NA	Non-complex	CKS
aml_ohsu_2018_13_00393	BeatAML	AML	89<4n>,XXY,-Y,-7,-12,+13,+13,-17,-21[2]/46,XY[18]	NA	Complex	Non-CKS
aml_ohsu_2018_13_00522	BeatAML	AML	46,XX,der(9)t(9;12)(p13;q13),del(11)(q21q25),der(12)add(12)(p11.2)add(12)(q13),del(13)(q14q22)[9]/46,idem,add(12)(q24.3),+13,-del(13)[9]/46,idem,add(17)(p13)[2]	NA	Complex	Non-CKS
aml_ohsu_2018_16_00048	BeatAML	AML	46,XX,del(3)(p25),add(6)(p22),t(10;12)(p15;q13)[5]/46,XX[18]	NA	Complex	Non-CKS
TCGA-AB-2849	TCGA	AML	47,XY,del(5)(q22q33),t(10;11)(p13~p15;q22~23),i(17)(q10)[3]/46,XY[17]	Non-complex	Complex	Non-CKS
TCGA-AB-2949	TCGA	AML	47,XY,+22[10]/47,XY,+8[7]/45,XY,del(3)(p21),del(4)(p12p15),-7,?dup(7)(q11.2q36)[3]	Non-complex	Complex	Non-CKS
K46	KUMC	AML	43,X,add(X)(q22),-5,-7,+11,der(11;15)(q10;q10),add(16)(q12),-17,-18,+mar[20]	NA	Complex	Non-CKS

AML Acute myeloid leukemia, *TCGA* The Cancer Genome Atlas, *KUMC* Korea University Medical Center, *NA* Not applicable, *CKS* Complex karyotype signature.
