# Supplementary material for: Identification of a Complex Karyotype Signature with Clinical Implications in AML and MDS-EB Using Gene Expression Profiling
Source: Cancers (Basel). 2023 Nov 4;15(21):5289. doi: 10.3390/cancers15215289 (PMC10648390; doi:10.3390/cancers15215289)
Supplement: Supplementary file 1 [file cancers-15-05289-s001.zip › Table S5.pdf]

Table S5. Patient characteristic of the TCGA cohort evaluated for clinical significance of complex karyotype.

	**Total (n=133)**	**CK (n=23)**	**Non-CK (n=110)**	***P***
Sex (%)				0.307
Male	65 (48.9)	9 (69.2)	56 (50.9)	
Female	58 (51.1)	4 (30.8)	54 (49.1)	
Age (years)				0.152
Median (range)	60.0 (18.0-88.0)	65.0 (18.0-81.0)	59.0 (21.0-88.0)	
WBC count (x 10^9/L)				**0.009**
Median (range)	16.0 (0.6-297.4)	9.9 (0.7-47.6)	24.5 (0.6-297.4)	
BM blasts (%)				**0.009**
Median (range)	72.0 (30.0-100.0)	58.0 (30.0-97.0)	75.0 (30.0-100.0)	
Risk stratification by 2022 ELN				**0.000**
Favorable	37 (27.8)	0 (0.0)	37 (33.6)	
Intermediate	39 (29.3)	0 (0.0)	39 (35.5)	
Adverse	48 (36.1)	23 (100.0)	25 (22.7)	
Not assessed	9 (6.7)	0 (0.0)	9 (8.2)	
Risk stratification by genetics				
FLT3-ITD				0.098
without	108 (81.2)	22 (95.7)	86 (78.2)	
with	25 (18.8)	1 (4.3)	24 (21.8)	
NA	0 (0.0)	0 (0.0)	0 (0.0)	
mutated *RUNX1*				0.171
without	118 (88.7)	23 (100.0)	95 (86.4)	
with	15 (11.3)	0 (0.0)	15 (13.6)	
NA	0 (0.0)	0 (0.0)	0 (0.0)	
Overall survival (months)				**0.021**
Median (range)	13.8 (0.0-118.1)	7.2 (0.0-95.5)	16.4 (0.1-118.1)	

TCGA The Cancer Genome Atlas, *WBC* White blood cell, *BM* Bone marrow, *ITD* Internal tandem duplication, *NA* Not applicable.
