# Supplementary material for: Identification of a Complex Karyotype Signature with Clinical Implications in AML and MDS-EB Using Gene Expression Profiling
Source: Cancers (Basel). 2023 Nov 4;15(21):5289. doi: 10.3390/cancers15215289 (PMC10648390; doi:10.3390/cancers15215289)
Supplement: Supplementary file 1 [file cancers-15-05289-s001.zip › Table S6.pdf]

Table S6. Patient characteristic of the BeatAML cohort evaluated for clinical significance of complex karyotype.

	**Total (n=120)**	**CK (n=22)**	**Non-CK (n=98)**	***P***
Sex (%)				0.736
Male	72 (60.0)	12 (54.5)	60 (61.2)	
Female	48 (40.0)	10 (45.5)	38 (38.8)	
Age (years)				0.620
Median (range)	61.0 (10.0-87.0)	63.0 (23.0-83.0)	61.0 (10.0-87.0)	
Hb (g/dL)				0.095
Median (range)	8.6 (3.7-16.1)	8.2 (5.7-11.0)	8.8 (3.7-16.1)	
WBC count (x 10^9/L)				**0.044**
Median (range)	20.8 (0.5-427.46)	9.3 (0.5-119.6)	22.9 (0.9-427.46)	
Platelet count (x 10^9/L)				0.386
Median (range)	47.0 (11.0-330.0)	33.0 (11.0-174.0)	47.0 (12.0-330.0)	
BM blasts (%)				**0.014**
Median (range)	65.0 (1.0-95.0)	45.0 (3.0-95.0)	72.2 (1.0-95.0)	
Risk stratification by 2022 ELN				**0.000**
Favorable	25 (20.8)	0 (0.0)	25 (25.5)	
Intermediate	32 (26.7)	0 (0.0)	32 (32.7)	
Adverse	55 (45.8)	22 (100.0)	33 (33.7)	
Not assessed	8 (6.7)	0 (0.0)	8 (8.2)	
Risk stratification by genetics				
FLT3-ITD				0.108
without	88 (73.3)	20 (90.9)	68 (69.4)	
with	23 (19.2)	1 (4.5)	22 (22.4)	
NA	9 (7.5)	1 (4.5)	8 (8.2)	
mutated *RUNX1*				0.653
without	95 (79.2)	19 (86.4)	76 (77.6)	
with	16 (13.3)	2 (9.1)	14 (14.3)	
NA	9 (7.5)	1 (4.5)	8 (8.2)	
Overall survival (months)				0.191
Median (range)	7.3 (0.07-45.92)	7.1 (0.43-40.69)	10.0 (0.07-45.92)	

AML Acute myeloid leukemia, *Hb* Hemoglobin, *WBC* White blood cell, *BM* Bone marrow, *ITD* Internal tandem duplication, *NA* Not applicable.
